# Multi-omics analysis of DNA replication-associated primase polymerase (PRIMPOL) in pan-cancer: a potential target for prognosis and immune response

**DOI:** 10.1186/s40001-023-01181-9

**Published:** 2023-06-30

**Authors:** Langmei Deng, Abhimanyu Thakur, Jinwu Peng, Liying Song, Zhilan Li

**Affiliations:** 1 Department of Emergency, The Third Xiangya Hospital, Central South University, Changsha, Hunan China; 2 Pritzker School of Molecular Engineering, Ben May, Department for Cancer Research, University of Chicago, Chicago, IL USA; 3 Department of Pathology, Xiangya Hospital, Central South University, Changsha, Hunan China; 4 Department of Pharmacy, The Third Xiangya Hospital, Central South University, Changsha, Hunan China; 5 Department of Pathology, Xiangya Changde Hospital, Changde, Hunan China

**Keywords:** PRIMPOL, Cancer, Immune infiltration, Prognosis, DNA damage response

## Abstract

**Background:**

It is critical to understand the mechanisms of human cancers in order to develop the effective anti-cancer therapeutic strategies. Recent studies indicated that primase polymerase (PRIMPOL) is strongly associated with the development of human cancers. Nevertheless, a systematic pan-cancer analysis of PRIMPOL remains to be further clarified.

**Method:**

Comprehensive multi-omics bioinformatics algorithms, such as TIMER2.0, GEPIA2.0 and cBioPortal, were utilized to evaluate the biological roles of PRIMPOL in pan-cancer, including the expression profiles, genomic alterations, prognostic values and immune regulation.

**Results:**

PRIMPOL was upregulated in glioblastoma multiforme and kidney renal clear cell carcinoma. The brain lower grade glioma patients with enhanced PRIMPOL expression displayed poor prognostic values. We also demonstrated the PRIMPOL's immunomodulating effects on pan-cancer as well as its genomic changes and methylation levels. The aberrant expression of PRIMPOL was linked to various cancer-associated pathways, including DNA damage response, DNA repair, and angiogenesis, according to single-cell sequencing and function enrichment.

**Conclusions:**

This pan-cancer analysis offers a thorough review of the functional roles of PRIMPOL in human cancers, suggesting PRIMPOL as a potentially important biomarker for the progression and immunotherapy of various cancers.

**Supplementary Information:**

The online version contains supplementary material available at 10.1186/s40001-023-01181-9.

## Introduction

Nowadays, multiple conventional treatment strategies have been developed for cancer patients; however, some patients eventually display the therapeutic resistance [1, 2]. Of late, immunotherapy has been increasingly used as a viable treatment option to fight cancers [3–6]. With the development of public databases, we could evaluate the functional roles of candidate genes in the clinical prognosis and immune response of cancer by pan-cancer analysis, thereby discovering new immunotherapeutic targets.

PRIMPOL (also known as CCDC111) is an enzyme with both primase and polymerase activity that is responsible for the efficient progression of replication forks, and replication stress is strongly associated with the development of cancer [7–9]. In addition, because DNA replication in cancer cells is usually uninhibited, DNA polymerase and DNA repair proteins have been used as therapeutic targets against some types of cancer [10]. Recently, a large number of studies have shown that PRIMPOL plays a key role in DNA replication, and changes in PRIMPOL activity may promote tumor formation [9, 11].For example, Quinet et al. found that in ovarian cancer cell UW, PRIMPOL depletion affects cell proliferation and cell viability in BRCA1-deficient cells [12]. Pilzecker's study showed that PRIMPOL had an anti-mutagenic activity in human invasive breast cancer, with a significant increase in point mutations in PRIMPOL defective tumors [13]. However, its detailed effects in different tumor types remain elusive.

Here, the PRIMPOL’s expression profile was compared and analyzed in tumor tissues with correlated normal tissues. As well, the genetic alterations and methylation levels of PRIMPOL, and its immunomodulating effects in a variety of tumors were also appraised. These comprehensive results could reveal the latent molecular mechanisms and biological functions of PRIMPOL in the occurrence, progression and clinical prognosis for patients with malignant tumors.

## Materials and methods

### The analysis of PRIMPOL expression profiles in pan-cancer

As shown in Additional file 7: Table S1, we used several bioinformatics algorithms to evaluate the PRIMPOL’s expression in pan-cancer, such as TIMER2.0 [14], GEPIA2.0 [15], UALCAN Platform [16] and Human Protein Atlas (HPA) [17]. The *p*-values < 0.05 and |Log_2_FC|≥ 1 were regarded as the selection conditions in GEPIA2.0. Using GEPIA2.0 database, we also analyzed the roles of PRIMPOL expression on patients’ tumor pathological stages. In addition, using UALCAN platform, we analyzed the methylation values and protein expression of PRIMPOL in pan-cancer.

### The patients’ prognosis analysis

Using GEPIA2.0 database, we obtained the survival data for the cancer patients with differentially expressed PRIMPOL, including overall survival (OS), disease-free survival (DFS), etc. The cutoff-low (50%) and cutoff-high (50%) were used as the threshold values to split the lowly expressed and highly expressed groups. The statistical differences were assessed by log-rank test.

### Genetic alteration evaluations

We used cBioPortal tool [18] to analyze the genetic changes of PRIMPOL in pan-cancer. In cBioPortal, the genetic changes mainly included gene mutations, deep deletion and amplification, etc.

### Immune evaluations

We used TIMER2.0 tool to consider the functional roles of PRIMPOL in the regulation of immunologic reactions in TCGA tumors. Several algorithms, including TIMER, TIDE and CIBERSORT, were used to evaluate the roles of PRIMPOL expression on the tumor-infiltrating B cells, fibroblasts, neutrophils, CD8 + T cells, myeloid dendritic cells, NK cells and T-regulatory cells (Tregs).

### Single-cell sequencing analysis

The heat map from the CancerSEA database [19] showed the biological function of PRIMPOL at the single-cell level. The machine learning algorithm, T-distributed stochastic neighbor embedding (t-SNE), was utilized to analyze the distribution of PRIMPOL expression in human malignancies.

### Gene function enrichment analysis

The STRING website was used to construct the PRIMPOL-associated molecule network [20, 21]. After then, the genes closely correlated with PRIMPOL in TCGA pan-cancer were obtained from GEPIA2.0 database. At last, the Kyoto Encyclopedia of Genes and Genomes (KEGG) mediated by PRIMPOL-associated genes we predicted by Xiantao XueShu [22].

### Statistical analysis

Student's T test was used to analyze the expression difference of PRIMPOL between tumor tissues and corresponding normal tissue. Spearman’s rank test was used to analyze the correlations between two groups. The functional values of PRIMPOL on the patients’ prognosis were confirmed by Kaplan–Meier plotter with log-rank test. The p values < 0.05 were deemed to be statistically significant.

## Results

### Gene expression profiles of PRIMPOL in pan-cancer

**Fig. 1:**
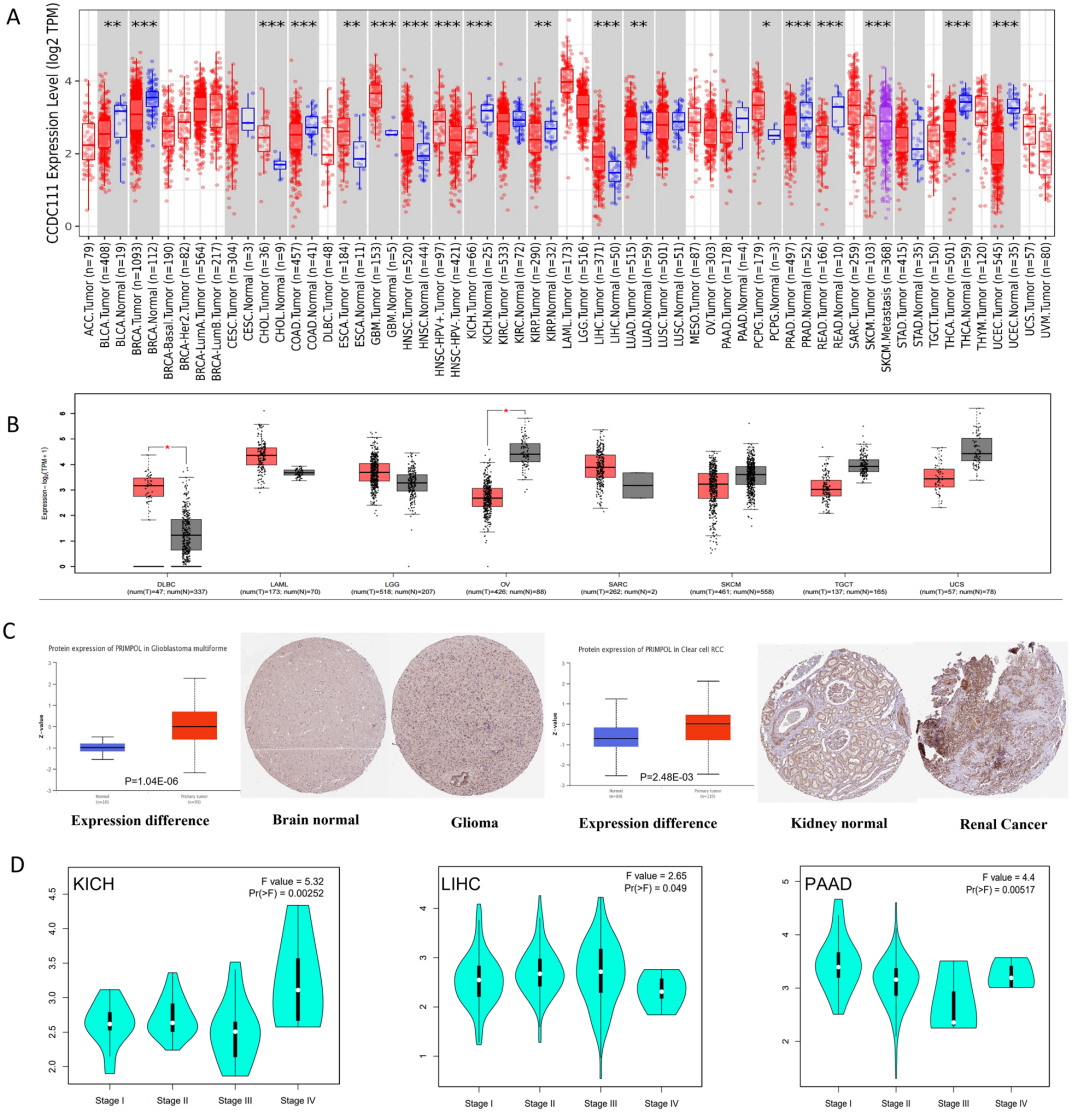
The expression levels of PRIMPOL in pan-cancer. **A** TIMER2.0 showed the PRIMPOL expression in TCGA cancers and the normal tissues. ***p < 0.001; **p < 0.01; *p < 0.05. **B** GEPIA2.0 revealed the expression level of PRIMPOL in the tumor and normal tissues. **C** UALCAN analysis results showed that the expression of PRIMPOL was upregulated in GBM and RCC compared with normal samples, and HPA showed the same results. **D** GEPIA2.0 displayed the relationship between PRIMPOL and pathological stages of KICH, LIHC and PAAD patients

At first step, through TIMER2.0 database, we investigated the expression profiles of PRIMPOL in human cancers. Figure 1A reveals that the PRIMPOL’s expression in BLCA, BRCA, COAD, KICH, KIRP, LUAD, PRAD, READ, UCEC and THCA are obviously downregulated than that in corresponding normal tissues. Conversely, in CHOL, ESCA, GBM, HNSC, LIHC, and PCPG, the PRIMPOL’s expression is obviously upregulated than the corresponding normal tissues. Given some normal tissues are missing in TCGA, we further used the combination of GTEx and TCGA datasets to confirm the differential expression of PRIMPOL between tumors and the corresponding normal tissues. The levels of PRIMPOL were significantly downregulated in OV; however, its levels were significantly upregulated in DLBC (Fig. 1B). In the other tumors, for example KIRC, PAAD and STAD, we did not obtain significant differences (Fig. 1A–B). In addition to its transcript levels, we also evaluated the PRIMPOL expression at the protein level using CPTAC from UALCAN tool. Figure 1C shows that PRIMPOL protein levels were significantly increased in GBM and KIRC tissues. Furthermore, we used GEPIA2 tool to investigate whether PRIMPOL expression is bound up with the pathological stage of tumors. Figure 1D reveals that there was an apparent interrelationship between PRIMPOL expression and the stages of KICH, LIHC and PAAD patients. However, there was no obvious relation between PRIMPOL expression and the stages of other tumors (Additional file 1: Fig. S1A–T). Overall, these findings collectively suggested the potential effects of PRIMPOL in cancer pathogenesis.

### The PRIMPOL expression on patients’ prognosis

**Fig. 2:**
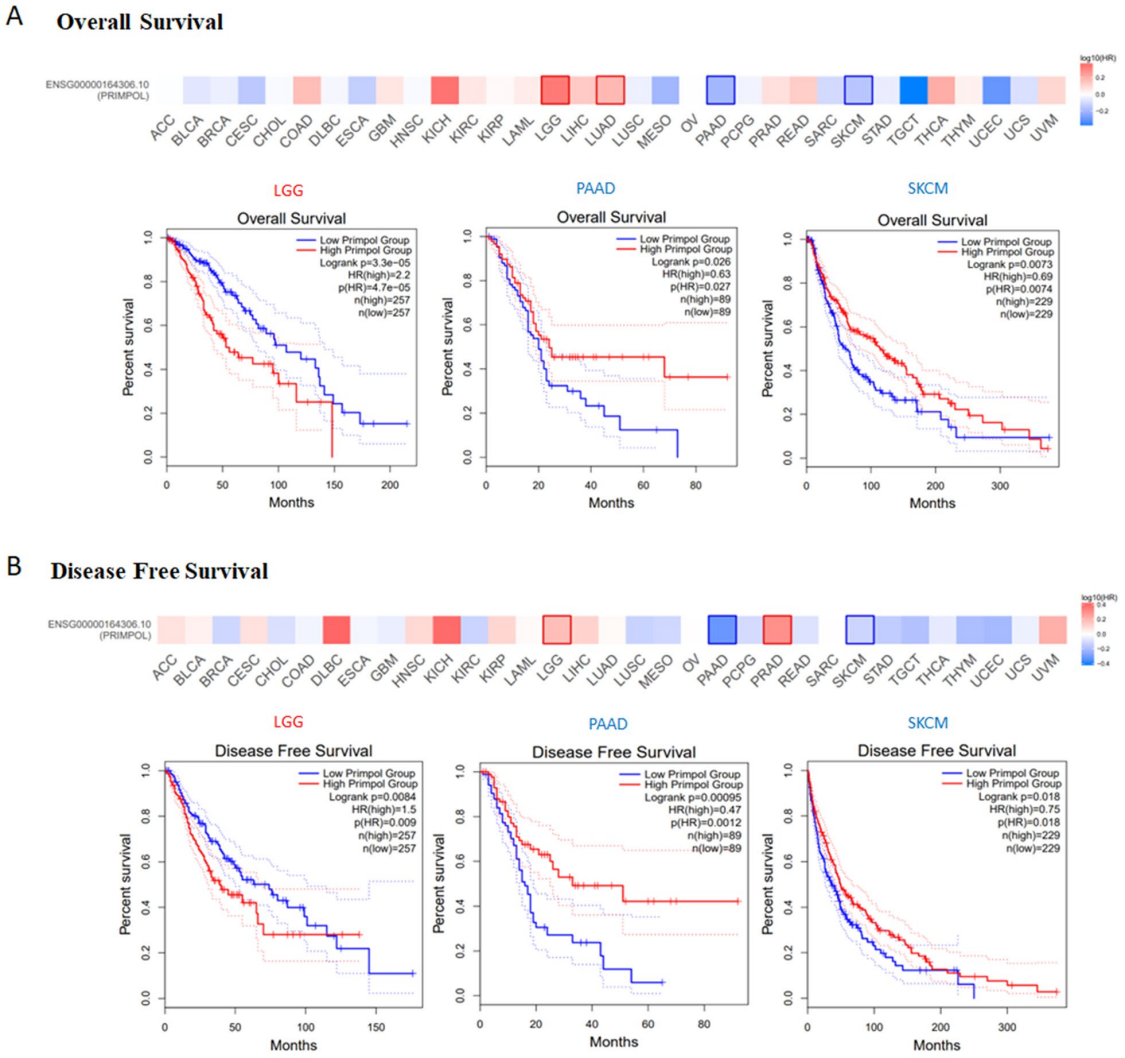
The effect of PRIMPOL on the prognosis of TCGA cancers. **A**, **B** GEPIA2.0 indicated the effects of PRIMPOL expression on the OS (**A)** and DFS (**B)** in TCGA cancers

Firstly, the patients were divided into two groups, PRIMPOL highly expressed group and PRIMPOL lowly expressed group. GEPIA2.0 database was used to evaluate the prognostic values of PRIMPOL in cancer patients. The findings indicated that patients with high levels of PRIMPOL in PAAD (*p* = 0.027) and SKCM (*p* = 0.0074) had a good OS. In contrast, patients with high levels of PRIMPOL in LGG have unfavorable OS (*p* = 4.7e-05) (Fig. 2A). Besides, we also analyzed the roles of PRIMPOL expression on patients’ DFS. As shown in Fig. 2B, the patients with high levels of PRIMPOL in PAAD (*p* = 0.0012) and SKCM (*p* = 0.018) have good DFS; however, the patients with high levels of PRIMPOL in LGG have unfavorable DFS (*p* = 0.009). Kaplan–Meier plotter survival analysis also showed that PRIMPOL expression was significantly associated with the prognosis of breast cancer and lung cancer (Additional file 2: Fig. S2).

### Genetic alteration analysis of PRIMPOL

**Fig. 3:**
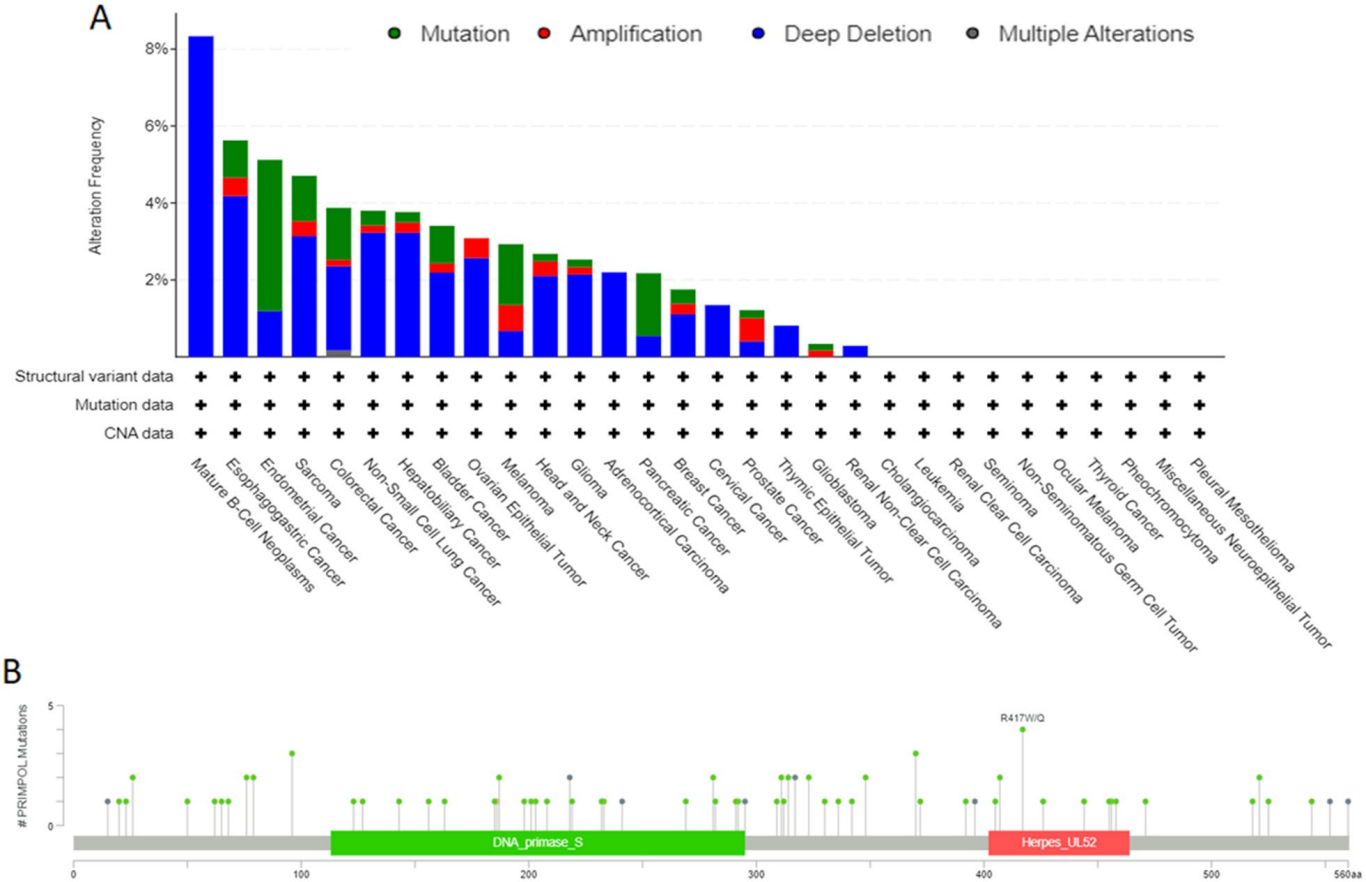
The mutation status of PRIMPOL across TCGA cancers. **A**, **B** cBioPortal displayed the mutation type, mutation frequency and mutation site of PRIMPOL in different cancers

**Fig. 4:**
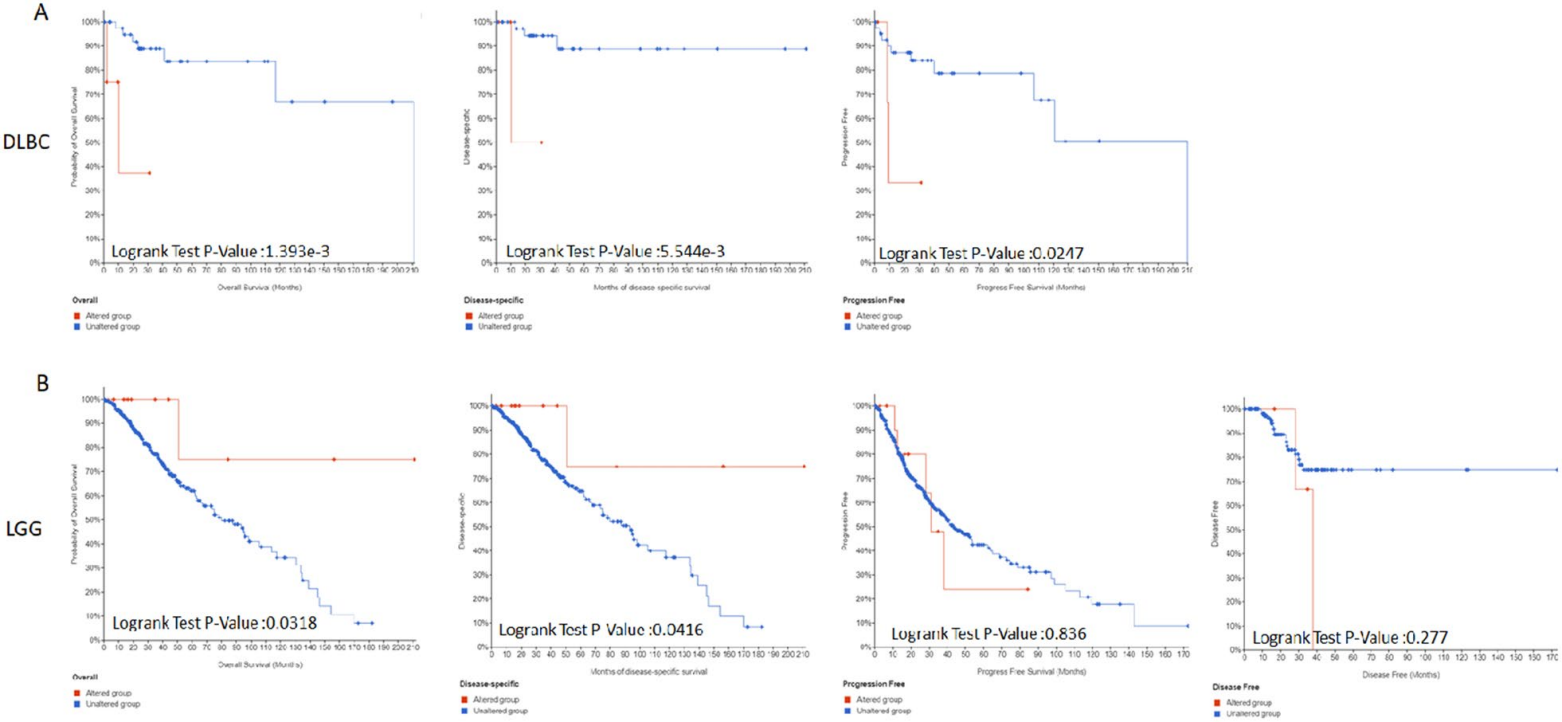
Effect of PRIMPOL alteration on prognosis of TCGA cancers. **A**, **B** cBioPortal showed survival graphs of PRIMPOL altered and unaltered groups in DLBC and LGG

Given the important effects of abnormal genomic changes in the malignant tumors [23, 24], we investigated the genetic alterations of PRIMPOL in various human tumors. Our study found that PRIMPOL "deep deletion" occur frequently in mature B-cell neoplasms and esophagogastric cancer. The incidence of PRIMPOL "mutation" is highest in endometrial cancer (Fig. 3A). Figure 3B indicates the promising roles of R417W/Q in cancer pathogenesis. In addition, the alteration of PRIMPOL was significantly associated with the prognosis of DLBC and LGG. Figure 4A shows that DLBC patients in the PRIMPOL altered group had worse OS (*p* = 1.393e-3), DSS (*p* = 5.544e-3) and PFS (*p* = 0.0247). However, patients in the PRIMPOL altered group had better OS (*p* = 0.0318) and DSS (*p* = 0.0416) in LGG, while PFS and DFS were not significantly different between the two groups (Fig. 4B). However, the alteration of PRIMPOL gene was not associated with the prognosis of other tumors (Additional file 3: Fig. S3A–D).

### The methylation values of PRIMPOL

**Fig. 5:**
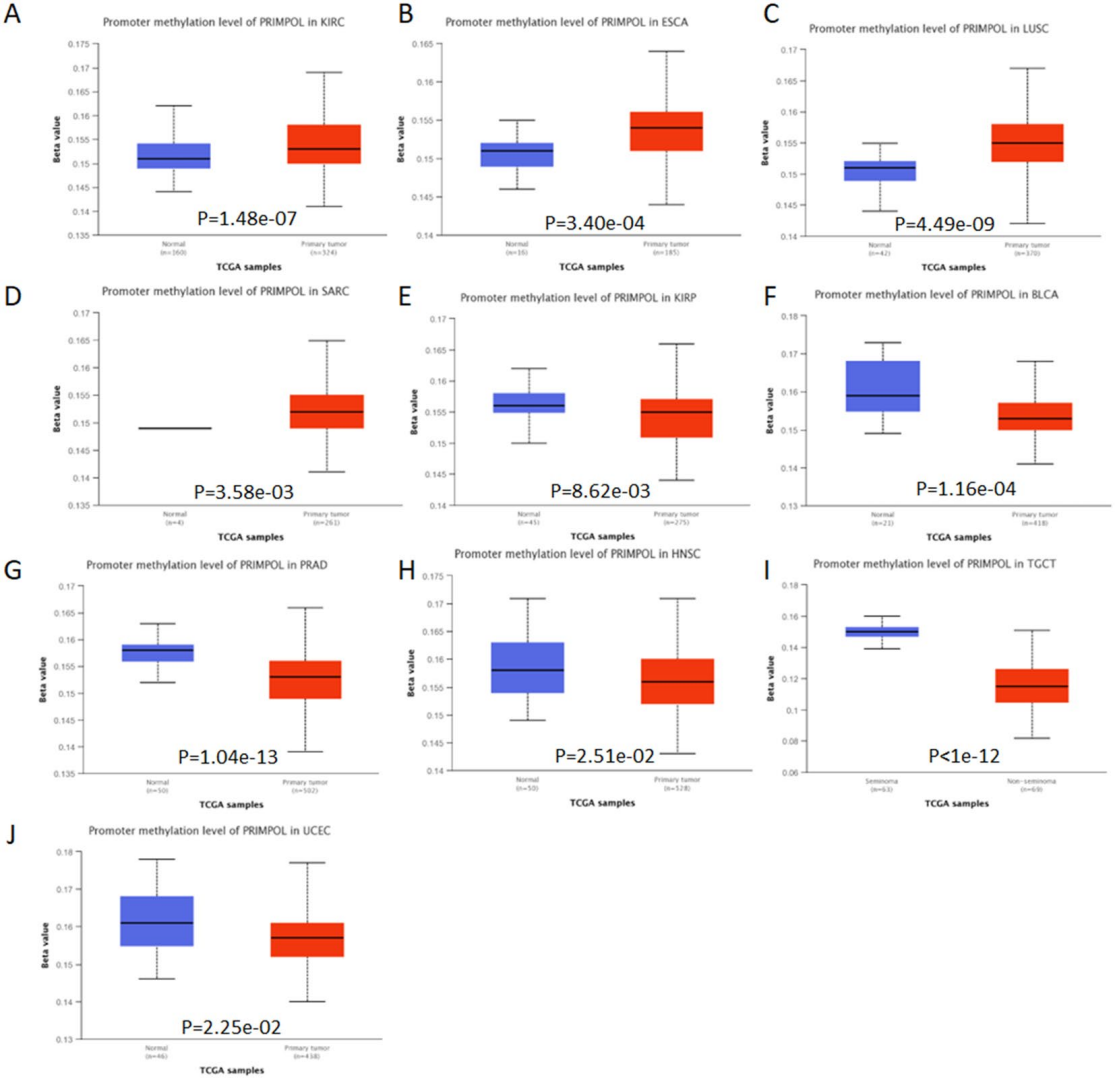
The methylation values of PRIMPOL in various tumors. **A**–**J** The UALCAN database showed the methylation values of PRIMPOL in multiple cancers

The changes of DNA methylation patterns have been proved to participate in the tumor development and prognosis, including hypermethylation and hypomethylation [25, 26]. Therefore, we make use of the UALCAN tool to figure out the methylation values of PRIMPOL in TCGA pan-cancer. These graphs showed that high methylation levels of PRIMPOL promoter in KIRC, ESCA LUSC and SARC (Fig. 5A–D), and low methylation levels of PRIMPOL promoter in KIRP, BLCA, PRAD, HNSC, TGCT and UCEC (Fig. 5E–J). However, in rest cancers, we did not find clear changes in the PRIMPOL promoter methylation level (Additional file 4: Fig. S4A–M). Meanwhile, we used the DiseaseMeth tool [27] to calculate the methylation levels of PRIMPOL in various tumors. We found that in BLCA, PRAD, and KIRP, PRIMPO methylation levels were lower than corresponding normal tissues, while in ESCA, LUSC, and SARC, PRIMPO methylation levels were higher than corresponding normal tissues. This is consistent with the conclusion we obtained using the UALCAN tool. In addition, we also found a significant decrease in PRIMPO methylation levels in LGG, ACC, READ, LAML, and LICH through DiseaseMeth analysis (Additional file 5: Figure S5A–K). Taken together, these results demonstrated that changes in PRIMPOL promoter methylation may be a key factor in the abnormal expression of PRIMPOL in cancer.

### Immune infiltration analysis data

**Fig. 6:**
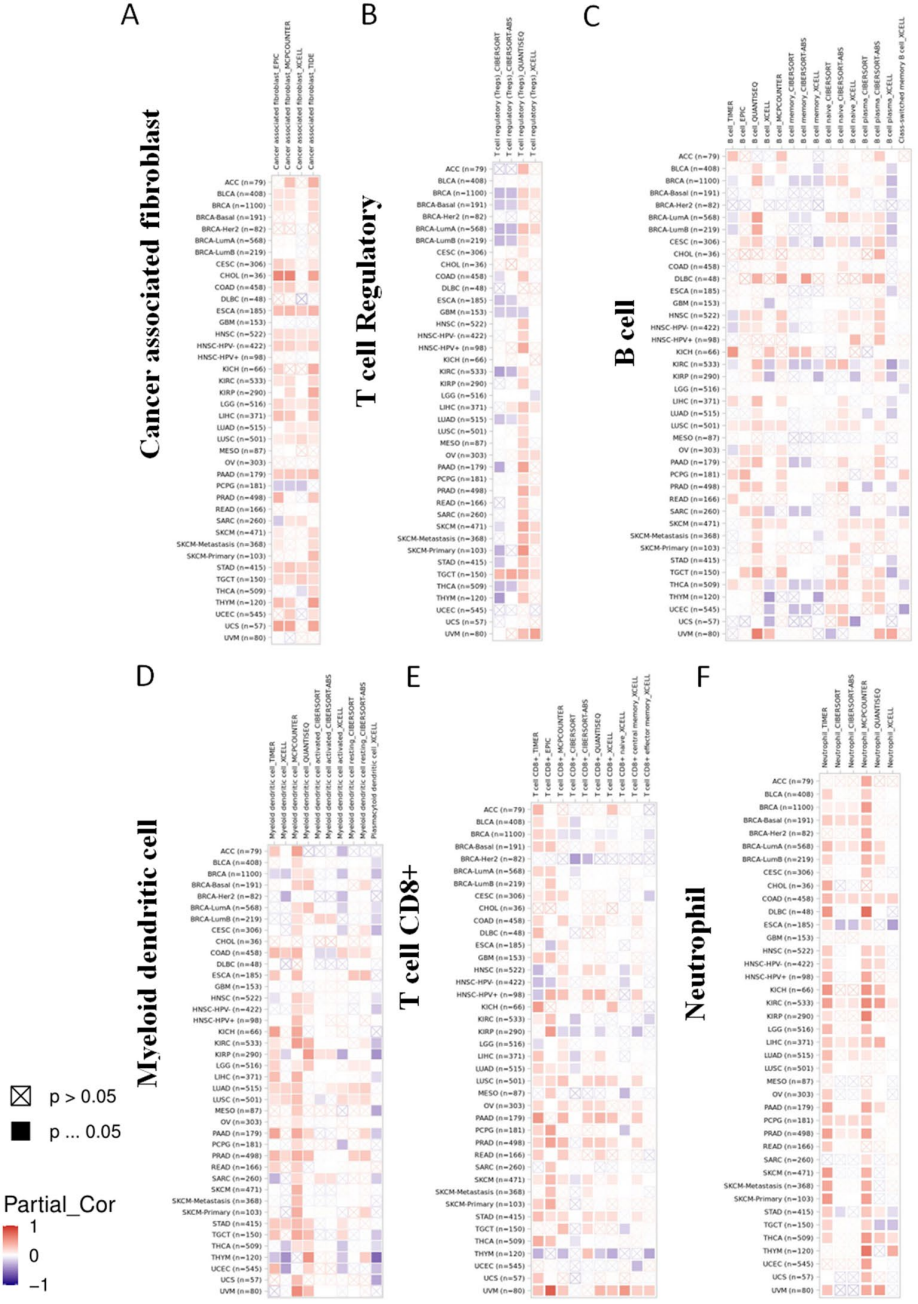
The relationship between PRIMPOL expression and different kinds of immune cells. **A**–**F** TIMER2.0 database analyzed the relationship between PRIMPOL expression and immune infiltration of cancer-associated fibroblasts (**A**), T-regulatory cells (**B**), B cells (**C**), myeloid dendritic cells (**D**), T cells CD8 + (**E**), and neutrophil (**F**). Multiple algorithms were used for each tumor, with blue representing a negative correlation between PRIMPOL expression and immune cells in the tumor and red representing a positive correlation

Of late, remarkable progresses have been seen in the field of tumor immunotherapy. The immunotherapeutic strategies have achieved remarkable clinical efficacy for the intervention of multiple cancers [28–30]. Accordingly, several algorithms, such as TIMER, EPIC and QUANTISEQ, were used to investigate the functional roles of PRIMPOL on the tumor-infiltrating immune cells. We discovered that the levels of PRIMPOL were positively interrelated with the penetration of cancer-associated fibroblasts (CAF) in a considerable number of tumors, including ESCA, HNSC, LGG, PAAD, STAD and TGCT (Fig. 6A). Besides, in TGCT, the levels of PRIMPOL were positively interrelated with the tumor infiltration of Tregs (Fig. 6B). The levels of PRIMPOL possessed a positive interrelation with the tumor penetration of B cells in LUSC (Fig. 6C). Besides, in PRAD and STAD, PRIMPOL expression levels were positively correlated with the tumor infiltration of myeloid dendritic cells (Fig. 6D). Moreover, the levels of PRIMPOL displayed the positively correlation with the tumor infiltration of CD8 + T cells in STAD (Fig. 6E). The levels of PRIMPOL were identified to be positively related with the tumor infiltration of neutrophil cells in COAD and KIRC (Fig. 6F). However, there was no clear correlation was discovered between the levels of PRIMPOL and tumor infiltration of NK cells (Additional file 6: Fig. S6). These results collectively illustrated that anomalously expressed PRIMPOL might go hand in hand with the immune infiltration in a variety of malignant tumors.

### The single-cell profiles of PRIMPOL in pan-cancer

**Fig. 7:**
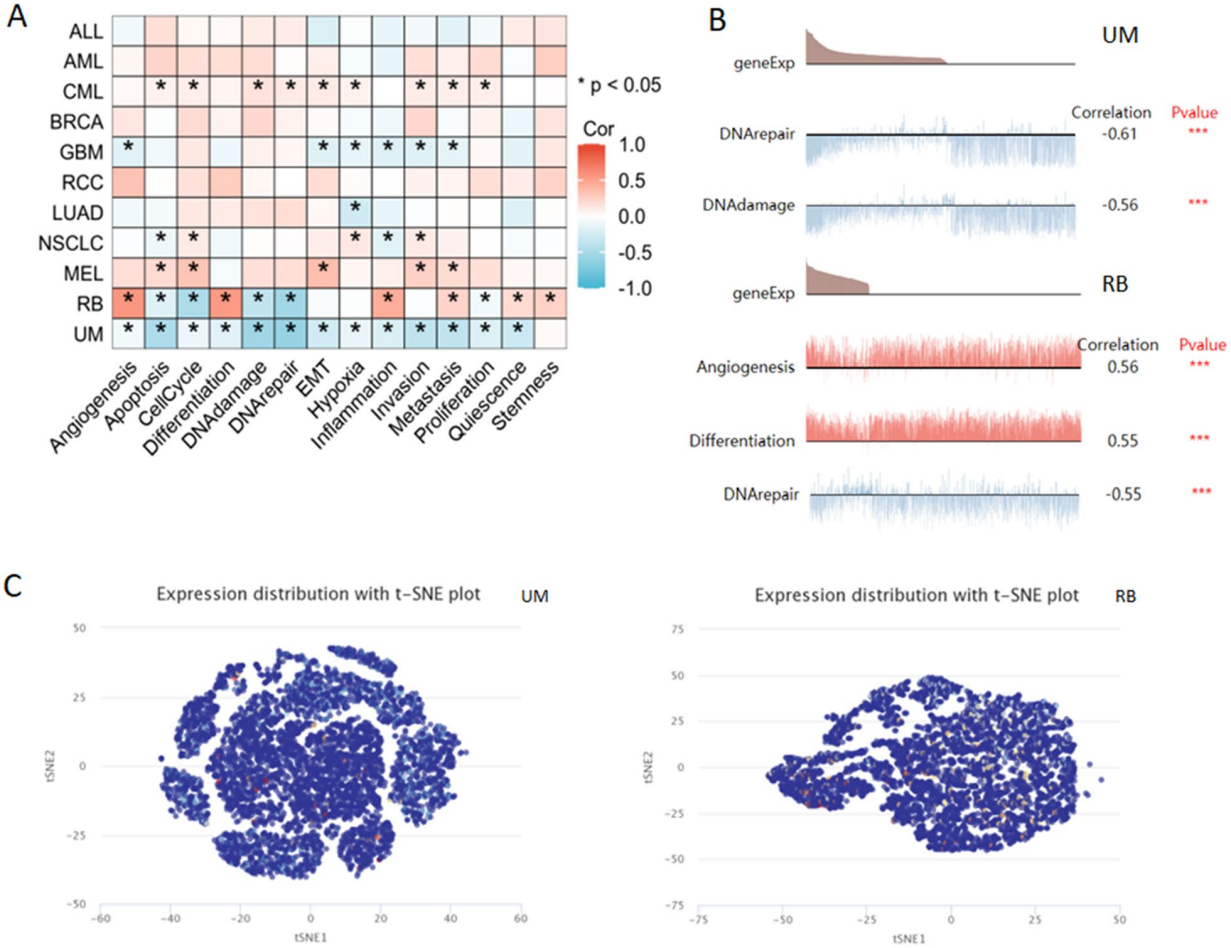
The expression levels of PRIMPOL at a single-cell sequence level. **A**, **B** CancerSEA displayed the relationship between the expression of PRIMPOL and different biological functions in tumors. **C** The t-SNE plot depict the distribution of PRIMPOL at the single-cell level in UM and RB tissues, every point represents a single cell, and the color of the point represents the expression level of PRIMPOL in the cell

The CancerSEA database was applied to study the single-cell expression distribution of PRIMPOL in pan-cancer. The results showed that PRIMPOL was significantly involved in several tumor-related signaling pathways in multiple tumors such as CML and GBM (Fig. 7A). PRIMPOL in RB had a significant positive correlation with angiogenesis, differentiation and inflammation, and a significant negative correlation with cell cycle and DNA repair. PRIMPOL in UM had a significant negatively correlated with multiple biological functions, including cell apoptosis, DNA damage response, DNA repair, invasion, metastasis and quiescence (Fig. 7A–B). In addition, Fig. 7C exhibits the single-cell distribution of PRIMPOL in UM and RB patients. Taken together, these findings suggested the important regulatory roles of PRIMPOL in the tumor biological pathways.

### The enrichment analysis of PRIMPOL-related molecules

**Fig. 8:**
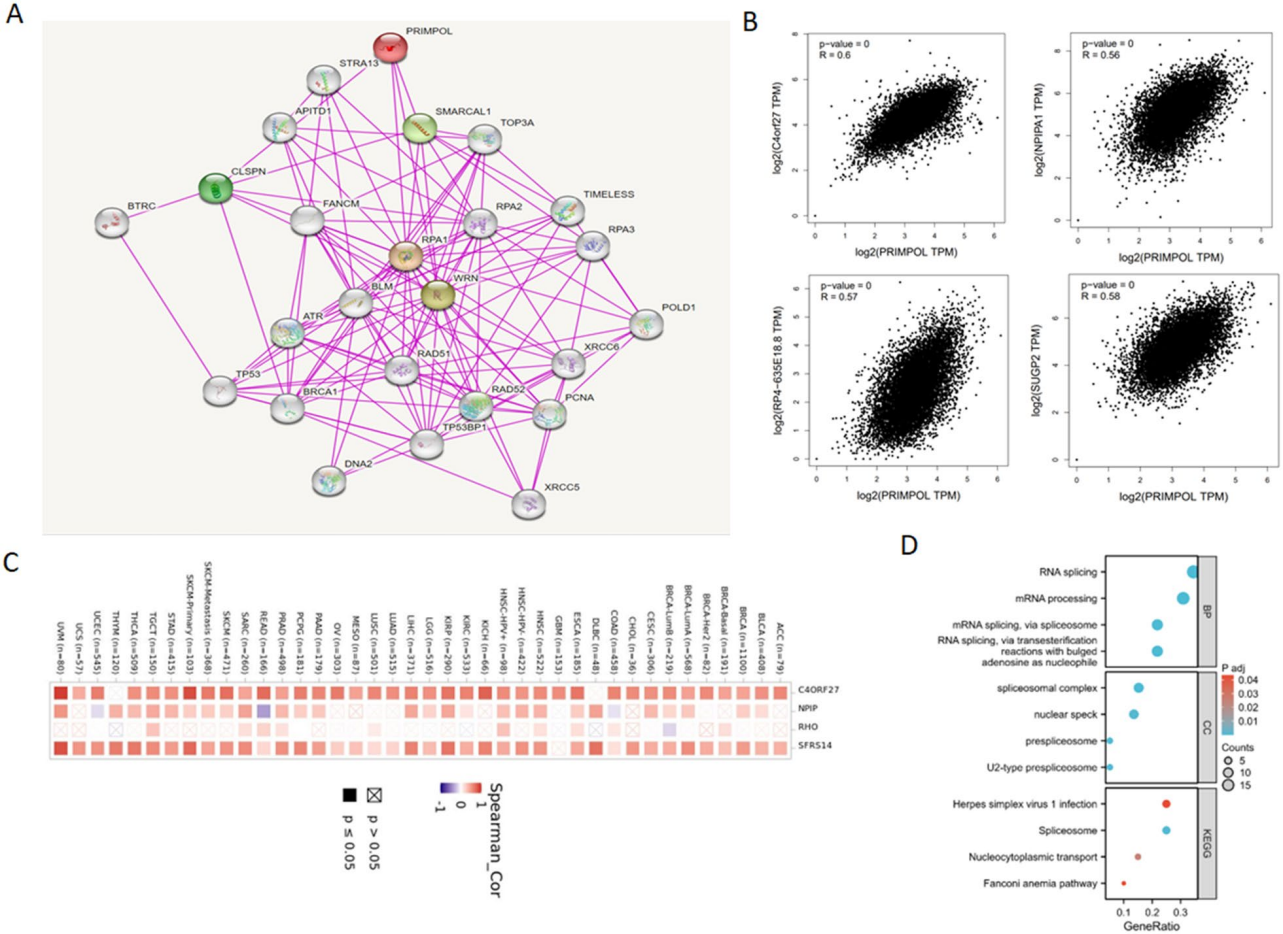
The functional enrichment analysis of PRIMPOL-related genes across TCGA cancers. **A** The interaction network of PRIMPOL-related genes derived from the STRING website. **B**, **C** GEPIA2.0 and TIMER2.0 showed four genes closely related to PRIMPOL expression. **D** The GO/KEGG analysis of PRIMPOL-related genes

In the last part, we used the enrichment analysis to evaluate the functional roles of PRIMPOL in cancers. We used the STRING website to investigate molecular biomarkers for PRIMPOL interactions (Fig. 8A). Then, GEPIA2.0 was used to obtain the first 100 PRIMPOL-related genes in pan-cancer (Additional file 8: Table S2). PRIMPOL expression was positively correlated with the levels of C4orf27, NPIPA1 (NPIP), RP4 (RHO) and SUGP2 (SFRS14) in pan-cancer (Fig. 8B). Afterwards, heat maps displayed that PRIMPOL was positively bound up with the afore-mentioned four genes in almost all malignant tumors, especially C4orf27 and SUGP2 (Fig. 8C). The GO and KEGG enrichment analysis indicated the potential regulatory roles of PRIMPOL-related genes in several gene expression-related biological pathways, like RNA splicing and mRNA processing (Fig. 8D).

## Discussion and conclusion

Several researches have suggested the crucial roles of aberrant PRIMPOL in the development and treatment of human cancers [12, 31]. Here, we utilized multiple bioinformatics platforms to conduct a pan-cancer analysis of PRIMPOL to illuminate its potential roles. The GTEx and TCGA datasets were used to evaluate the expression profiles of PRIMPOL in tumors. In addition, aberrated expressed PRIMPOL displayed the effect on the patients’ prognosis in PAAD, SKCM and LGG. Furthermore, in patients with KICH, LIHC and PAAD, PRIMPOL levels were significantly related to the pathological stages. Taken together, these findings collectively suggested that PRIMPOL might serve as a promising prognostic target for cancer patients. Our results indicate a regulatory effect between the survival and staging of PAAD patients, even though the underlying mechanism remains to be clarified. In the future, in vitro and in vivo experiments could be conducted to investigate the specific mechanisms and biological functions of PRIMPOL in the occurrence and development of PAAD.

Recent studies have the important regulatory roles of CAFs in tumor immune response, which could affect the anti-tumor activity of tumor-infiltrating immune cells [32, 33]. In solid tumors, CAFs could recruit and stimulate immunosuppressive cells, and induce the formation of dense extracellular matrix (ECM), consequently affecting the anti-tumor immunity [34]. In our study, the results indicated that PRIMPOL was evidently associated with the tumor-infiltrating CAFs in multiple malignancies. These findings suggest that abnormally expressed PRIMPOL might regulate the tumor infiltration of CAF and influence the anti-tumor immunity. Neutrophils are a kind of immune cells with special biological properties against infections and inflammation [35]. Neutrophil extracellular traps (NETs), released upon neutrophil activation, could prevent the action of cytotoxic lymphocytes, thereby directly or indirectly promoting the tumor growth and progression [36]. In our study, PRIMPOL is involved in the regulation of neutrophils in a variety of tumors, such as BLCA, BRCA, KIRC and LIHC. As the main effector cells of immunity, B cells can inhibit the progression of cancer by kill cancer cells directly or indirectly [37]. In this study, a significant correlation between PRIMPOL and the tumor-infiltrating B cells have been displayed in certain tumors. In addition, PRIMPOL was also found to be associated with the tumor infiltration of CD8 + T cells and other immune cells. These data collectively demonstrated the vital roles of aberrant PRIMPOL in the regulation of tumor immune microenvironment and immunotherapeutic response.

To sum up, we clarified that the functional effects of PRIMPOL on the patients’ clinical prognosis, pathological stages and immune response in various malignant tumors. Overall, these comprehensive results would be helpful to clarify the detailed roles of PRIMPOL in tumor progression, prognosis and treatment. Due to the limitations of bioinformatics technology, further in vivo or in vitro experiments are needed to confirm the biological roles of PRIMPOL in human cancers.

## Supplementary Information

**Additional file 1: Fig. S1** GEPIA2.0 displayed the relationship between PRIMPOL and pathological stages of TCGAcancers.

**Additional file 2: Fig. S2** Kaplan-Meier Plotter demonstrated the effect of PRIMPOL expression on the prognosis ofbreast cancer and lung cancer.

**Additional file 3: Fig. S3** cBioPortal showed survival graphs of PRIMPOL altered and unaltered groups in TCGAcancers.

**Additional file 4: Fig. S4** The UALCAN database showed the methylation values of PRIMPOL in multiple cancers.

**Additional file 5: Fig. S5** The DiseaseMeth database showed the methylation values of PRIMPOL in multiplecancers.

**Additional file 6: Fig. S6** TIMER2.0 database analyzed the relationship between PRIMPOL expression and NKcells.

**Additional file 7: Table S1** Bioinformatics platforms that are employed to analyze the role of PRIMPOL in pan-cancer.

**Additional file 8: Table S2** The first 100 PRIMPOL-related genes in pan-cancer obtained from the GEPIA2.0database

## Data Availability

All data of this study are included in the article or supplementary information, which can be obtained from the corresponding author.

## Abbreviations

PRIMPOL: Primase-polymerase
HPA: Human Protein Atlas
OS: Overall survival
DFS: Disease-free survival
TCGA: The Cancer Genome Atlas
KEGG: Kyoto Encyclopedia of Genes and Genomes
BLCA: Bladder urothelial carcinoma
BRCA: Breast invasive carcinoma
COAD: Colon adenocarcinoma
KICH: Kidney chromophobe
KIRP: Kidney renal papillary cell carcinoma
LUAD: Lung adenocarcinoma
PRAD: Prostate adenocarcinoma
READ: Rectum adenocarcinoma
UCEC: Uterine corpus endometrial carcinoma
THCA: Thyroid carcinoma
CHOL: Cholangiocarcinoma
ESCA: Esophageal carcinoma
GBM: Glioblastoma multiforme
HNSC: Head and neck squamous cell carcinoma
LIHC: Liver hepatocellular carcinoma
PCPG: Pheochromocytoma and paraganglioma
OV: Ovarian serous cystadenocarcinoma
DLBC: Lymphoid neoplasm diffuse large B-cell lymphoma
KIRC: Kidney renal clear cell carcinoma
PAAD: Pancreatic adenocarcinoma
STAD: Stomach adenocarcinoma
SKCM: Skin cutaneous melanoma
LGG: Brain lower grade glioma
DSS: Disease-specific survival
PFS: Progression-free survival
RB: Retinoblastoma
UM: Uveal melanoma
CAF: Cancer-associated fibroblasts
