# Supplementary figures and images for: Multi-omics analysis of DNA replication-associated primase polymerase (PRIMPOL) in pan-cancer: a potential target for prognosis and immune response
Source: Eur J Med Res. 2023 Jun 30;28:207. doi: 10.1186/s40001-023-01181-9 (PMC10314441; doi:10.1186/s40001-023-01181-9)

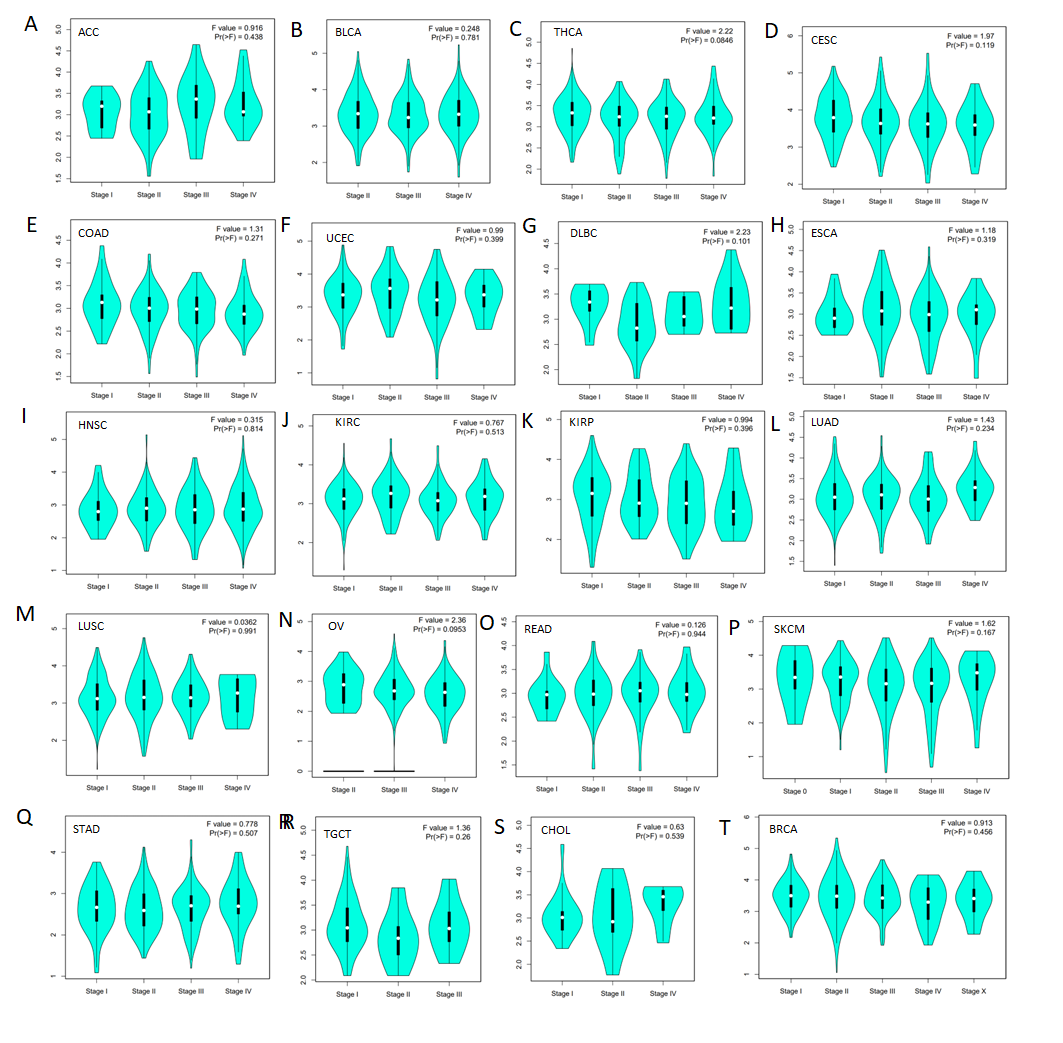

Supplement: Supplementary file 1 — Additional file 1: Fig. S1 GEPIA2.0 displayed the relationship between PRIMPOL and pathological stages of TCGAcancers. [file 40001_2023_1181_MOESM1_ESM.tif]

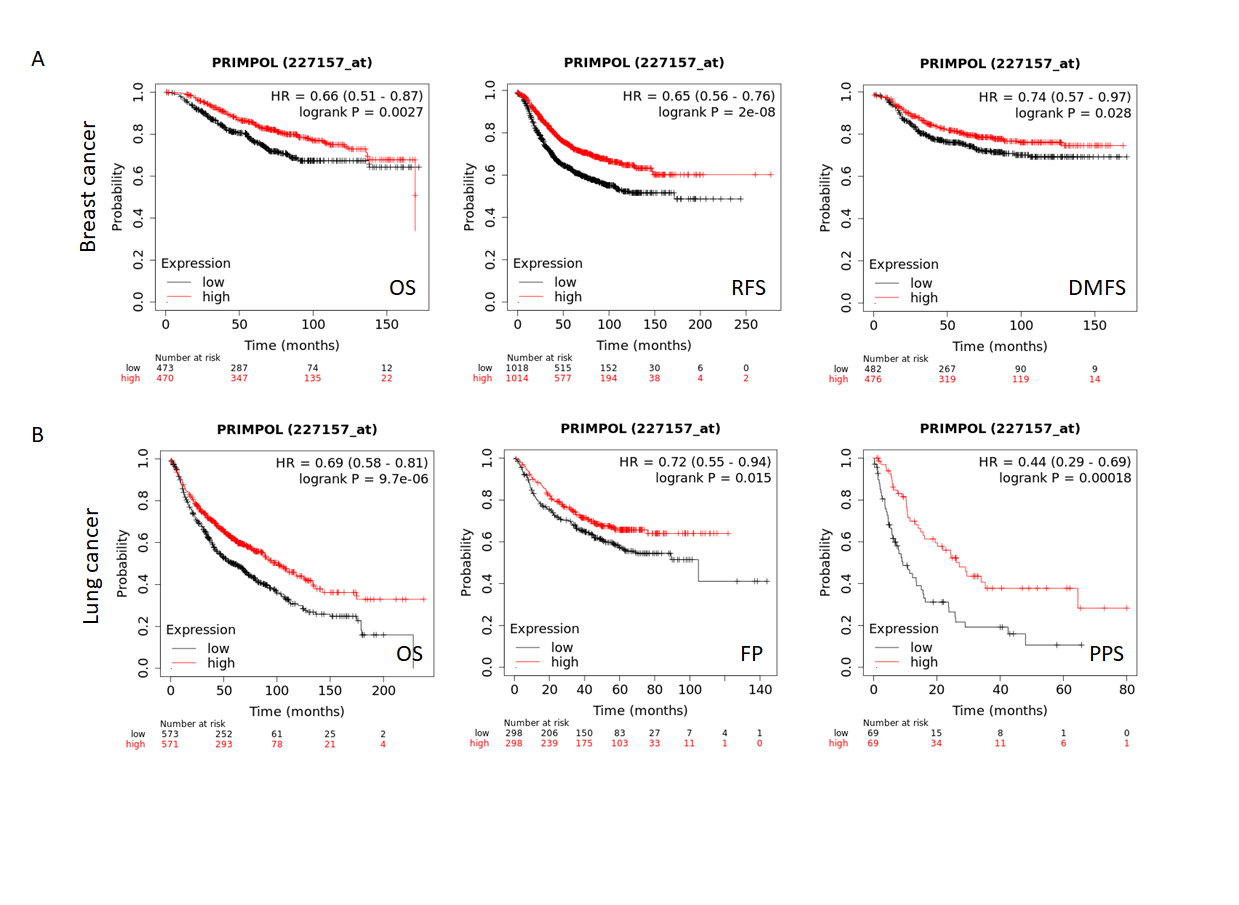

Supplement: Supplementary file 2 — Additional file 2: Fig. S2 Kaplan-Meier Plotter demonstrated the effect of PRIMPOL expression on the prognosis ofbreast cancer and lung cancer. [file 40001_2023_1181_MOESM2_ESM.tif]

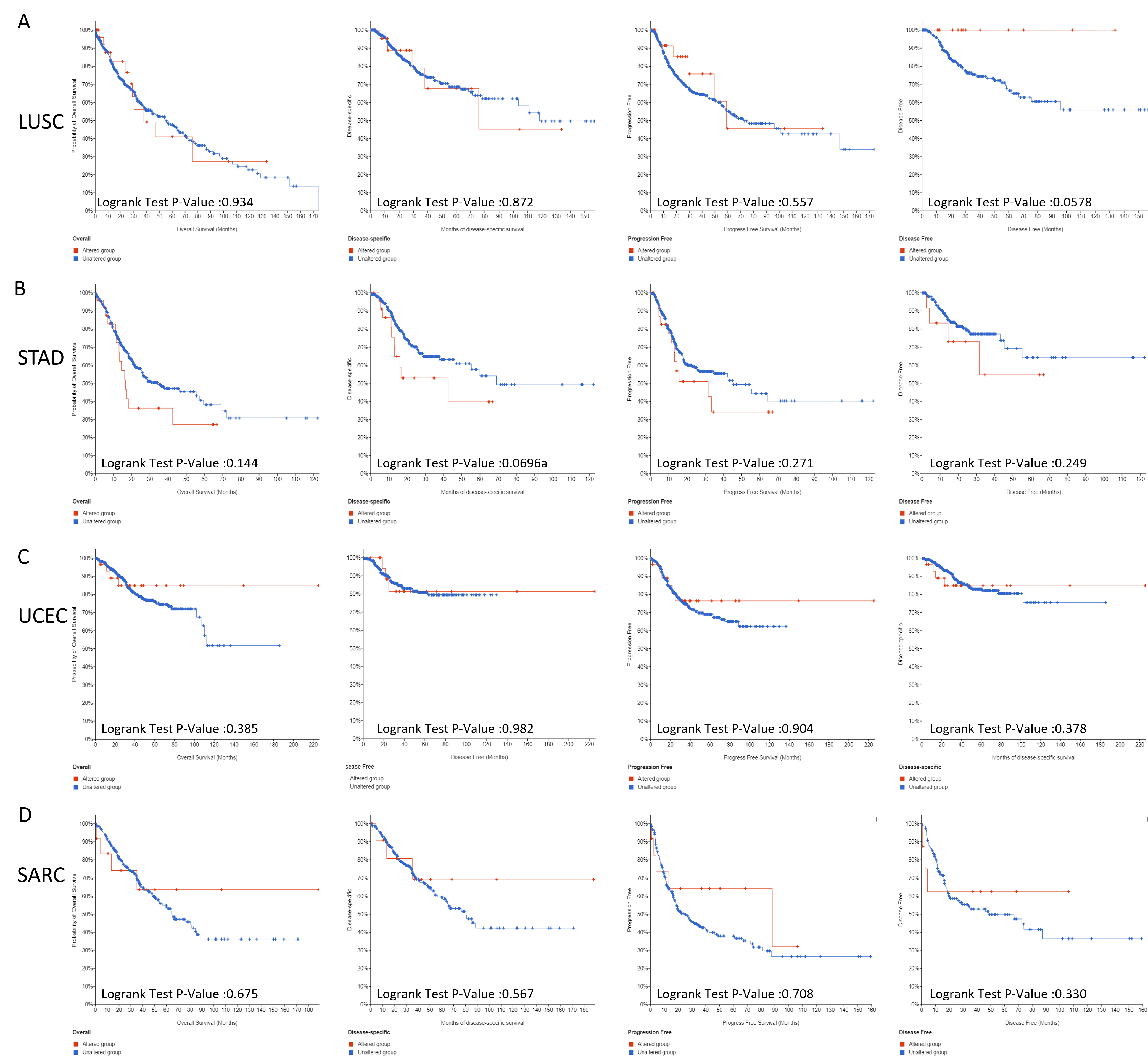

Supplement: Supplementary file 3 — Additional file 3: Fig. S3 cBioPortal showed survival graphs of PRIMPOL altered and unaltered groups in TCGAcancers. [file 40001_2023_1181_MOESM3_ESM.tif]

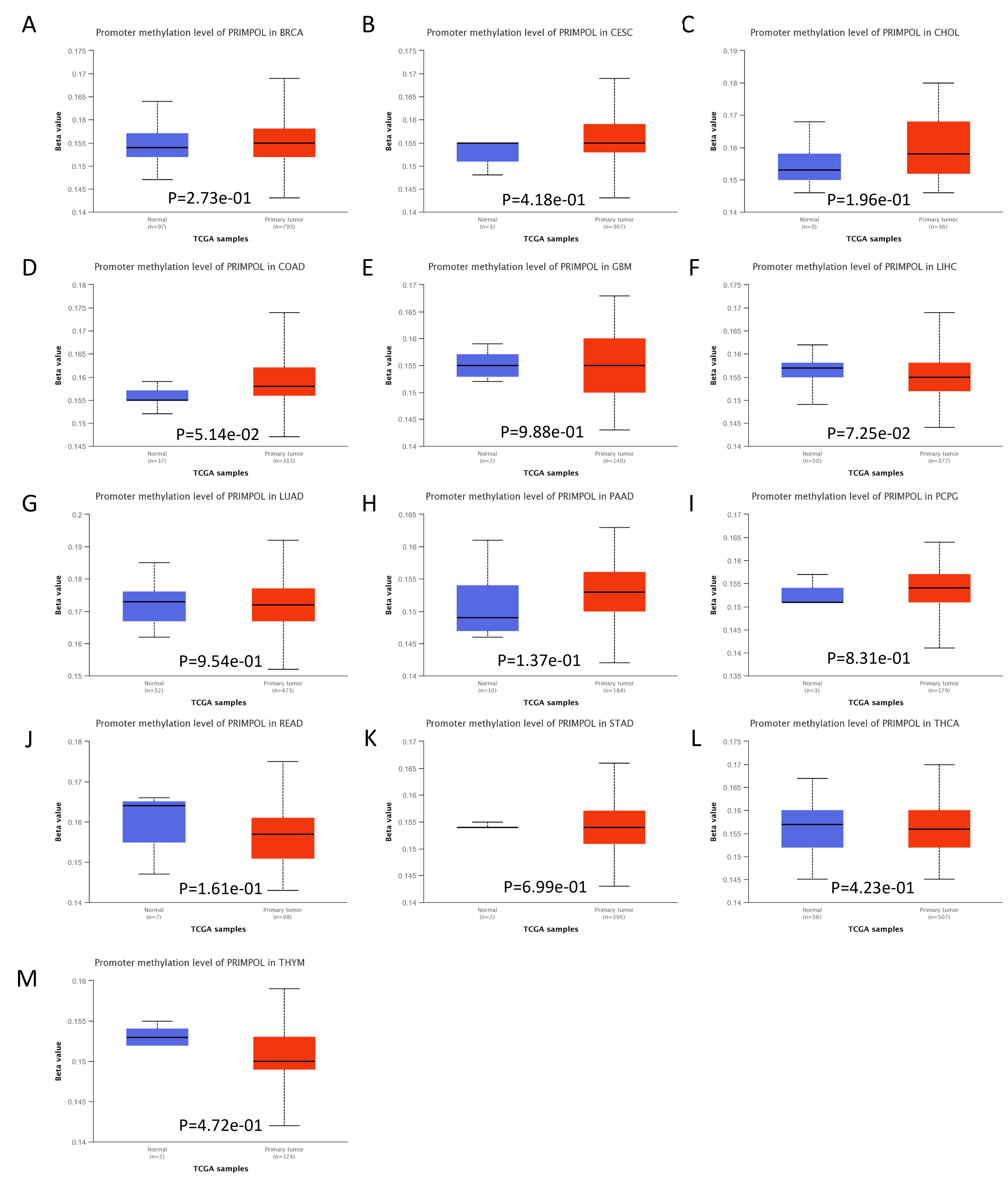

Supplement: Supplementary file 4 — Additional file 4: Fig. S4 The UALCAN database showed the methylation values of PRIMPOL in multiple cancers. [file 40001_2023_1181_MOESM4_ESM.tif]

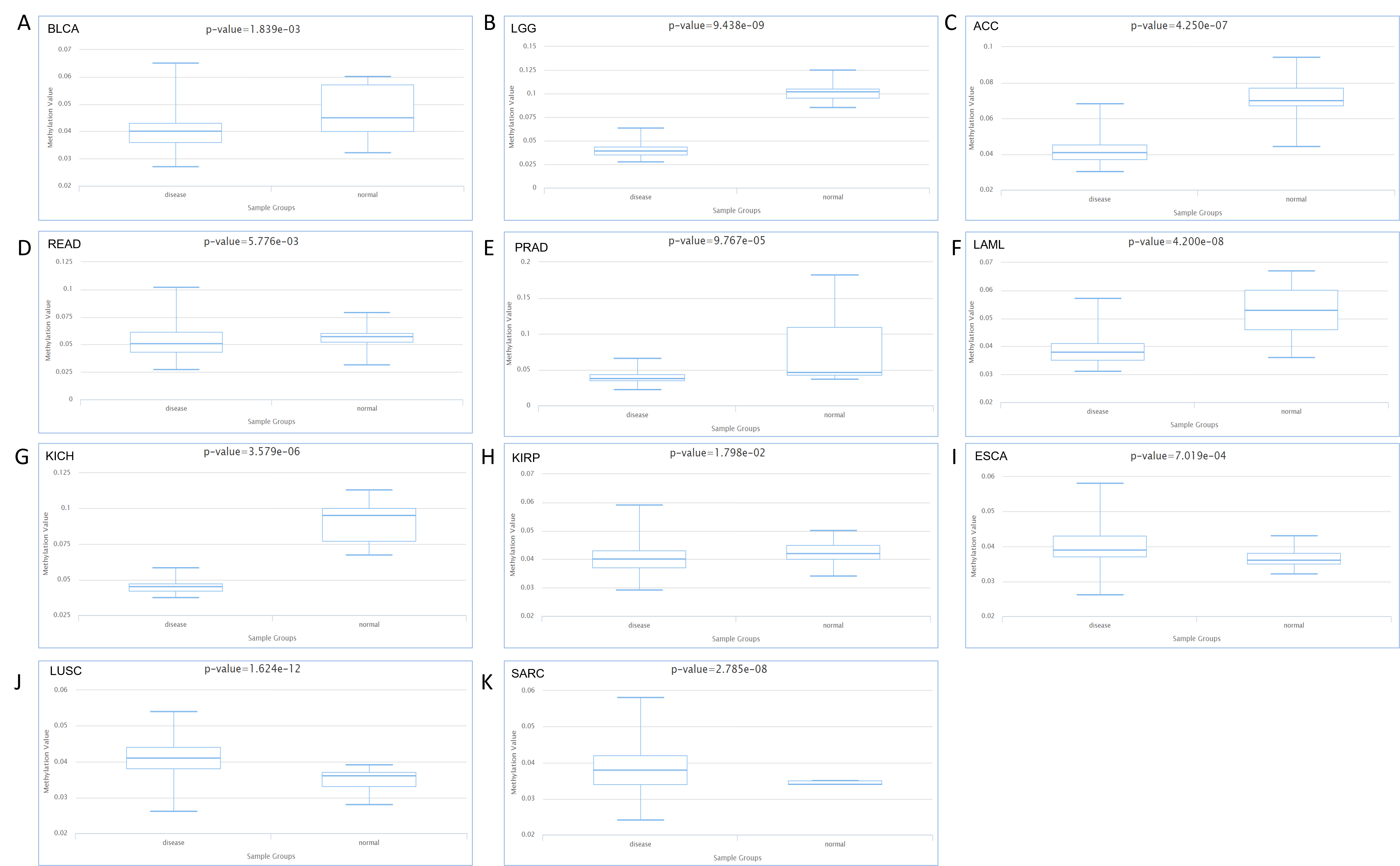

Supplement: Supplementary file 5 — Additional file 5: Fig. S5 The DiseaseMeth database showed the methylation values of PRIMPOL in multiplecancers. [file 40001_2023_1181_MOESM5_ESM.tif]

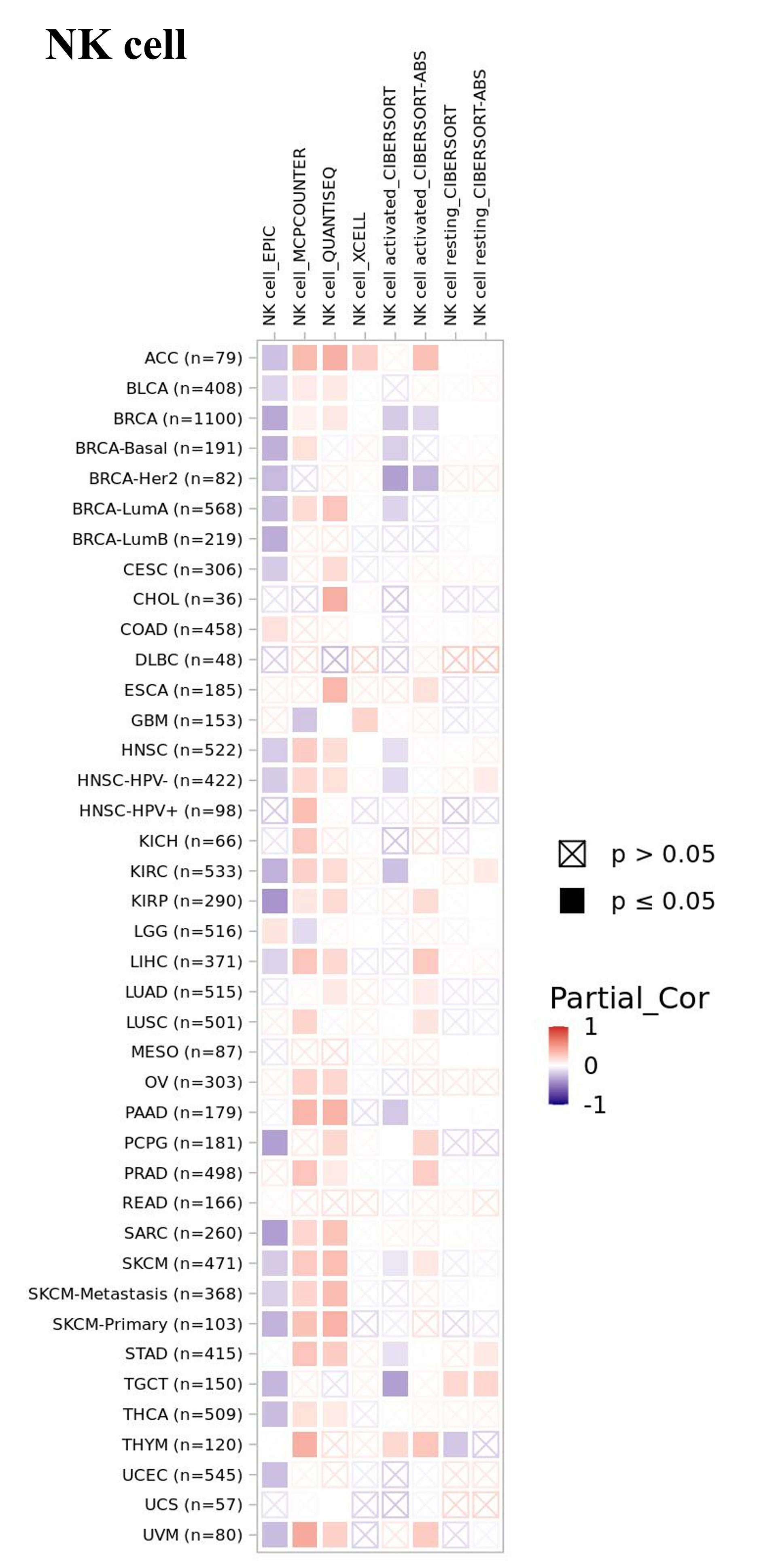

Supplement: Supplementary file 6 — Additional file 6: Fig. S6 TIMER2.0 database analyzed the relationship between PRIMPOL expression and NKcells. [file 40001_2023_1181_MOESM6_ESM.tif]
