# Supplementary material for: Multi-omics analysis of DNA replication-associated primase polymerase (PRIMPOL) in pan-cancer: a potential target for prognosis and immune response
Source: Eur J Med Res. 2023 Jun 30;28:207. doi: 10.1186/s40001-023-01181-9 (PMC10314441; doi:10.1186/s40001-023-01181-9)
Supplement: Supplementary file 7 — Additional file 7: Table S1 Bioinformatics platforms that are employed to analyze the role of PRIMPOL in pan-cancer. [file 40001_2023_1181_MOESM7_ESM.docx]

Table S1. Bioinformatics platforms that are employed to analyze the role of PRIMPOL in pan-cancer.

| Bioinformatics platform | Website link | References | Year |  |
| --- | --- | --- | --- | --- |
| TIMER2.0 | http://timer.cistrome.org/ | 14 | 2020 | |
| GEPIA2.0 | http://gepia2.cancer-pku.cn/ | 15 | 2021 | |
| cBioPortal | https://www.cbioportal.org/ | 18 | 2014 | |
| UALCAN | https://ualcan.path.uab.edu/ | 16 | 2022 | |
| DiseaseMeth | http://biobigdata.hrbmu.edu.cn/diseasemeth/index.html | 27 | 2017 | |
| Human Protein Atlas | https://www.proteinatlas.org/ | 17 | 2011 | |
| Xiantao XueShu | <https://www.xiantao.love/products> | 22 | 2023 | |
| CancerSEA | http://biocc.hrbmu.edu.cn/CancerSEA/ | 19 | 2019 | |
| STRING | https://string-db.org | 20 | 2019 | |
